# The Joint Role of Thyroid Function and Iodine Status on Risk of Preterm Birth and Small for Gestational Age: A Population-Based Nested Case-Control Study of Finnish Women

**DOI:** 10.3390/nu11112573

**Published:** 2019-10-25

**Authors:** Alexandra C. Purdue-Smithe, Tuija Männistö, Griffith A. Bell, Sunni L. Mumford, Aiyi Liu, Kurunthachalam Kannan, Un-Jung Kim, Eila Suvanto, Heljä-Marja Surcel, Mika Gissler, James L. Mills

**Affiliations:** 1 Epidemiology Branch, Division of Intramural Population Health Research, *Eunice Kennedy Shriver* National Institute of Child Health and Human Development, National Institutes of Health, Bethesda, MD 20892, USA; alexandra.purdue-smithe@nih.gov (A.C.P.-S.); mumfords@mail.nih.gov (S.L.M.); 2 Northern Finland Laboratory Centre NordLab, 90120 Oulu, Finlan; Tuija.Mannisto@Nordlab.fi (T.M.); Eila.Suvanto@ppshp.fi (E.S.); 3 Department of Clinical Chemistry, University of Oulu, 90120 Oulu, Finland; 4 Medical Research Center Oulu, Oulu University Hospital and University of Oulu, 90120 Oulu, Finland; 5 Finnish Institute for Health and Welfare, 00290 Helsinki, Finland; mika.gissler@thl.fi; 6 Ariadne Labs, Brigham and Women’s Hospital, Harvard T.H. Chan School of Public Health, Boston, MA 02115, USA; griffith.bell@gmail.com; 7 Harvard T.H. Chan School of Public Health, Department of Health Policy and Management, Boston, MA 02115, USA; 8 Biostatistics and Bioinformatics Branch, Division of Intramural Population Health Research, *Eunice Kennedy Shriver* National Institute of Child Health and Human Development, National Institutes of Health, Bethesda, MD 20892, USA; liua@mail.nih.gov; 9 Wadsworth Center, New York State Department of Health, Albany, NY 12201, USA; kurunthachalam.kannan@health.ny.gov (K.K.); changetm2011@gmail.com (U.-J.K.); 10 Biobank Borealis of Northern Finland, Oulu University Hospital, 90120 Oulu, Finland; helja-marja.surcel@thl.fi; 11 Faculty of Medicine, University of Oulu, 90120 Oulu, Finland; 12 Karolinska Institute, 17177 Stockholm, Sweden

**Keywords:** iodine, thyroid hormones, thyroglobulin, thyroid stimulating hormone, pregnancy, preterm birth, small for gestational age

## Abstract

Normal maternal thyroid function during pregnancy is essential for fetal development and depends upon an adequate supply of iodine. Little is known about how iodine status is associated with preterm birth and small for gestational age (SGA) in mildly iodine insufficient populations. Our objective was to evaluate associations of early pregnancy serum iodine, thyroglobulin (Tg), and thyroid-stimulating hormone (TSH) with odds of preterm birth and SGA in a prospective, population-based, nested case-control study from all births in Finland (2012–2013). Cases of preterm birth (*n* = 208) and SGA (*n* = 209) were randomly chosen from among all singleton births. Controls were randomly chosen from among singleton births that were not preterm (*n* = 242) or SGA (*n* = 241) infants during the same time period. Women provided blood samples at 10–14 weeks’ gestation for serum iodide, Tg and TSH measurement. We used logistic regression to estimate odds ratios (ORs) and 95% confidence intervals (CIs) for preterm birth and SGA. Each log-unit increase in serum iodide was associated with higher odds of preterm birth (adjusted OR = 1.19, 95% CI = 1.02–1.40), but was not associated with SGA (adjusted OR = 1.01, 95% CI = 0.86–1.18). Tg was not associated with preterm birth (OR per 1 log-unit increase = 0.87, 95% CI = 0.73–1.05), but was inversely associated with SGA (OR per log-unit increase = 0.78, 95% CI = 0.65–0.94). Neither high nor low TSH (versus normal) were associated with either outcome. These findings suggest that among Finnish women, iodine status is not related to SGA, but higher serum iodide may be positively associated with preterm birth.

## 1. Introduction

Normal maternal thyroid function during pregnancy is essential for fetal development [1]. Hypothyroidism is associated with adverse pregnancy outcomes including pregnancy loss, preeclampsia, and preterm birth, as well as cognitive deficiencies and cretinism in the offspring [2]. Iodine, found in fish, eggs, dairy products, and iodized salt [3], plays an essential role in the production of thyroid hormones. Thyroid hormone production is regulated by the hypothalamic-pituitary-thyroid axis via thyroid-stimulating hormone (TSH) and requires the iodination of thyroglobulin (Tg) in the follicular lumen of thyrocytes [1]. Pregnant women are especially vulnerable to iodine deficiency due to fetal dependency on the maternal iodine supply and to a lesser extent, hemodilution, increased renal clearance of inorganic iodide, and estrogen-stimulated production of Tg, which collectively necessitate higher iodine intake [4].

Despite increased risk of iodine deficiency during pregnancy, even in developed countries [5,6], and ample data indicating that thyroid dysfunction is associated with adverse neonatal and obstetric outcomes, relatively few studies have evaluated how iodine status during pregnancy is related to preterm birth and infants being born small for gestational age (SGA), and the results are conflicting [7,8,9,10,11,12,13]. Importantly, previous studies were conducted in populations with high prevalence of other nutritional deficiencies and concurrent illnesses or included mostly iodine sufficient pregnant women [9,12]. Several of these studies also lacked information on thyroid hormones and therefore were unable to evaluate whether associations of iodine with preterm birth and SGA may be different among potentially hypo-or hyperthyroid women [9,10,11,13]. Additionally, prior studies used a single spot urinary iodine measurement to classify iodine status, which has been shown to have high intraindividual variability reflective of recent dietary intake, seasonal variation, urine dilution, and circadian rhythmicity [13,14,15,16]. Although urine iodine concentrations are useful for assessing the iodine status of whole populations, serum iodide may be less sensitive to recent dietary intake and may, therefore, better reflect individual long-term and bioavailable iodine status [17,18,19], reducing potential for misclassification. 

The aim of the present study was to evaluate associations of serum iodide concentrations and thyroid hormones indicative of iodine status (i.e., Tg and TSH) with risk of preterm birth and SGA among pregnant Finnish women, a population considered to be mildly iodine deficient, but with relatively low prevalence of other nutritional deficiencies [3].

## 2. Materials and Methods 

### 2.1. Study Population

We conducted a population-based, nested case-control study within the Finnish Maternity Cohort (FMC), using the Finnish Medical Birth Register (MBR) to ascertain pregnancy and perinatal outcome data. Beginning in 1983, the FMC has collected more than 2 million serum samples from more than 950,000 pregnant women living in Finland, which reflects ~98% coverage of the pregnant population. Blood samples were collected to screen for hepatitis B, human immunodeficiency virus (HIV), syphilis, and rubella antibodies. The samples were drawn in general between 10 and 14 weeks gestation at local maternity care units and sent to the prenatal serology laboratory of the Finnish Institute for Health and Welfare in Oulu. There, sera were separated by centrifugation, screening analyses were performed and the remaining serum (1–3 mL) was stored at −25 °C.

Biochemical data were linked to clinical data from the MBR via unique personal identification numbers given to all Finnish citizens and residents at birth or at time of permanent residence. The MBR includes data on all live births and stillbirths in Finland with a birth weight ≥500 g or a gestational age at birth ≥22 gestational weeks. Maternal data collected by the MBR includes age, height and weight, socioeconomic status based on self-reported occupation, marital status, pregnancy history, smoking status, and other factors. Data collected on infants included sex, gestational age at birth, and birth height and weight.

Women gave written informed consent for their samples to be used for research purposes. This study was approved by the steering committee of the FMC, the ethical review boards of the Northern Ostrobothnia Hospital District and the Finnish Institute for Health and Welfare, Oulu, Finland, and the Office of Human Subjects Research, National Institutes of Health, Bethesda, MD, USA (#13459).

### 2.2. Case and Control Ascertainment

We randomly selected 200 cases of preterm birth (defined as a live birth <37 weeks gestation) and 250 potential controls from among all singleton births in Finland between 2012 and 2013 with available serum samples in the FMC. Because the 250 potential controls were randomly selected without regard to case/control status, 8 control pregnancies were delivered preterm and were thus reclassified as cases. After reclassification, the final analytic sample included 208 cases and 242 controls. Similarly, we randomly selected 200 cases of SGA (defined as birthweight <10^th^ percentile for gestational age), using the same control group. Nine control pregnancies were SGA and reclassified as such, resulting in 209 SGA cases and 241 controls. 

### 2.3. Measurement of Iodide, Thyroglobulin, and Thyroid-Stimulating Hormone

Details regarding the iodide measurement in this study population have been published previously and are provided in Appendix A [20]. Briefly, serum samples were thawed at room temperature, vortexed, and transferred to polypropylene tubes. Samples were pretreated, centrifuged, and analyzed by high-performance liquid chromatography (Alliance 2695 HPLC) coupled with electrospray triple-quadrupole mass spectrometry (Micromass, ESI–MS/MS; Waters Corporation, Milford, MA, USA). Identification and quantification of ^18^O-labeled-perchlorate, ^13^C-labeled-thiocyante, and iodide was performed using electrospray negative ionization (ESI-) and multiple reaction monitoring. Serum Tg and TSH concentrations were measured using a commercial immunoassay (Siemens AG, Munich, Germany), as they have been shown to be reliable markers of thyroid function in pregnant women [21]. The intra- and inter-assay coefficients of variation for Tg were <8% and <12%, respectively, and for TSH <5% and <5%, respectively. 

### 2.4. Statistical Analysis

Characteristics of the cases and controls were compared using t-tests for continuous variables and χ^2^ tests for categorical variables. Equivalent non-parametric tests were used where appropriate. For analyses evaluating continuous exposures, serum iodide, Tg, and TSH values were normalized by log-transformation. Participants were divided into quartiles of iodide and Tg based on the distribution of these biomarkers in the control group. For TSH, we categorized participants as having high TSH (>3.1 and >3.5 mIU/L in the 1st and 2nd trimesters, respectively), normal TSH (0.1–3.1 and 0.2–3.5 in the 1st and 2nd trimesters, respectively), or low TSH (<0.1 and <0.2 mIU/L in the 1st and 2nd trimesters, respectively), according to previously defined reference ranges for this population [22].

Using logistic regression, we estimated unadjusted odds ratios (ORs) and 95% confidence intervals (CIs) for preterm birth and SGA according to each biomarker. We then estimated adjusted ORs and 95% CIs adjusting for maternal age, maternal body mass index (BMI), socioeconomic status, smoking status, parity, and marital status. Covariates were selected for inclusion in multivariable models based on directed acyclic graphs [23]. Individuals with missing data on BMI (N = 7 for preterm birth and N = 3 for SGA) were dropped from multivariable analyses.

We also examined possible non-linear associations of iodide with preterm birth and SGA non-parametrically utilizing restricted cubic spline models. In these models, we specified 3 knots and evaluated the individual spline term contributions to the model fit and overall test for nonlinearity.

In sensitivity analyses, we excluded women with conditions associated with medically-indicated preterm birth (i.e., chronic hypertension, gestational hypertension, gestational diabetes, pre-existing diabetes, thyroid disease) to determine whether these conditions may have influenced the results. All analyses were run using SAS, version 9.4 (SAS Institute Inc., Cary, NC, U.S.). 

## 3. Results

### 3.1. Descriptive Characteristics

Characteristics of preterm birth and SGA cases and controls at blood draw are presented in Table 1. For preterm birth, cases and controls were similar with regard to maternal age, BMI, smoking status, marital status, parity, gravidity, and socioeconomic status (SES). Preterm birth cases and controls were also similar in terms of gestational age at blood draw (10.9 weeks vs. 10.8 weeks). As expected, cases were more likely to have chronic hypertension (10% vs. 3%), preeclampsia (30% vs. 3%), Type 1 or 2 diabetes (13% vs. 1%), and gestational diabetes (38% vs. 22%), compared to controls. For SGA, cases had lower BMI (24 vs. 25 kg/m^2^), gravidity (1.3 vs. 1.4) and parity (0.8 vs. 1.1) than controls, and were also more likely to be smokers (52% vs. 37%). SGA cases were also more likely than controls to have preeclampsia during the pregnancy (16% vs. 4%). The Spearman rank correlation coefficients for each biomarker were as follows: iodide and Tg, r_s_ = 0.02 (*P* = 0.54); iodide and TSH, r_s_ = 0.001 (*P* = 0.97); and Tg and TSH, r_s_ = −0.13 (*P* < 0.001).

### 3.2. Serum Iodine, Thyroid Hormones, and Preterm Birth

In unadjusted models, each 1 log-unit increase in serum iodide was positively associated with preterm birth (unadjusted OR = 1.22, 95% CI = 1.04–1.42) (Table 2). After adjusting for age, BMI, smoking, and other factors, the positive association persisted (adjusted OR = 1.19, 95% CI = 1.02–1.40), and was robust to the exclusion of women with conditions related to preterm birth (OR = 1.29, 95% CI = 1.06–1.58). In unadjusted models evaluating quartiles of iodide, the OR for preterm birth comparing women with high (quartile 4) versus moderate (quartiles 2+3) serum iodide was 1.32 (95% CI = 0.86–2.04). The OR for preterm birth comparing low (quartile 1) versus moderate (quartiles 2 + 3) serum iodide was 0.75 (95% CI = 0.46–1.22). In adjusted models, the ORs for preterm birth comparing women with high (quartile 4) and low (quartile 1) versus those with moderate (quartiles 2 and 3) serum iodide were 1.22 (95% CI = 0.78–1.93) and 0.76 (95% CI = 0.46–1.26), respectively. In spline analyses, no signifcant departure of linearity was observed for serum iodide and preterm birth (P for non-linearity > 0.05). No associations were observed for Tg and TSH and preterm birth (adjusted OR = 0.87, 95% CI = 0.73–1.05 and OR = 0.97, 95% CI = 0.80–1.19, respectively). 

### 3.3. Serum Iodide, Thyroid Hormones, and Small for Gestational Age

Serum iodide was not associated with odds of having an SGA infant in unadjusted or adjusted models. (Table 3) For example, the unadjusted OR for each log-unit increase in serum iodide was 0.99 (95% CI = 0.85–1.14). Similarly, in unadjusted models, the OR for SGA comparing high (quartile 4) and low (quartile 1) versus moderate (quartiles 2 + 3) serum iodide was 1.15 (95% CI = 0.74–1.80) and 1.05 (95% CI = 0.85–1.14), respectively. Adjustment for age, BMI, and other factors resulted in similar findings (OR per 1 log-unit increase in serum iodide = 1.01, 95% CI = 0.86–1.18). In unadjusted models, log-transformed Tg was inversely associated with SGA (unadjusted OR = 0.84, 95% CI = 0.71-0.99). This inverse association was somewhat stronger after adjustment in multivariable analyses (adjusted OR = 0.78, 95% CI = 0.65–0.94). Likewise, in adjusted models, the OR comparing high (quartile 4) versus low (quartile 1) Tg was 0.45 (95% CI = 0.25–0.79). TSH was not associated with SGA (high versus normal OR = 0.56, 95% CI = 0.14–2.22; low versus normal OR = 1.11, 95% CI = 0.44–2.79). Spline analyses revealed no significant departures of linearity for serum iodide and SGA (P for non-linearity > 0.05).

## 4. Discussion

In this population-based, nested case-control study, we found that neither low- nor high-serum iodide was associated with SGA, and some suggestion that higher serum iodide may be associated with increased risk of preterm birth. Levels of TSH, which were largely within the normal range, indicated a mostly euthyroid population. Tg, which is generally higher during periods of both iodine insufficiency and extreme excess [24,25,26], was inversely associated with risk of having an SGA infant, but was not associated with preterm birth. Levels of TSH were not associated with either outcome, although very few women had values considered to be above or below the normal range in this population. Collectively, our findings suggest that iodine insufficiency, within the range observed among pregnant Finnish women, likely does not play a role in preterm birth or SGA, but that higher serum iodide may be associated with increased risk of preterm birth.

To date, only a handful of studies have evaluated associations of iodine status with preterm birth and SGA, and data are mixed [7,8,9,10,11,12,13]. A recent prospective study of pregnant women in the UK reported U-shaped relationships between urinary iodine concentrations and risks of preterm birth and SGA, with elevated risks observed among women with urinary iodine concentration (UIC) <50 and ≥250 µg/L versus those with UIC in the 150–249 µg/L group; however, confidence intervals were wide and included the null value [10]. Another prospective study of pregnant women in the UK with low obstetrical risk reported a borderline significant increased risk of having an SGA infant with increasing UIC, but no association with preterm birth [13]. Similarly, in a largely iodine deficient population of pregnant women in Thailand, insufficient (UIC <150 µg/L) versus sufficient/excess (UIC ≥150 µg/L) iodine status was associated with increased risks of both preterm birth and low birth weight (LBW) infants [9]. Similar associations were observed among pregnant Chinese women, in which UIC <50 µg/L versus ≥50 µg/L was associated with a non-significant higher risk of LBW and SGA [12]. In another prospective study of Chinese women, both iodine insufficiency and excess were inversely associated with fetal femur length, a measure of fetal growth [11]. Among Spanish women, lower rates of SGA were observed among women with UIC 100–149 µg/L compared to those with <50 µg/L [8], but no associations of UIC with birth weight, SGA, or preterm birth were observed in another study of Spanish women [8]. In our study population of Finnish women, we found that higher serum iodide was positively associated with preterm birth, but neither high nor low serum iodide was associated with SGA.

Only a few previous studies have evaluated associations of Tg and TSH with preterm birth and SGA. In two prospective studies of pregnant Spanish women, high TSH, which is indicative of hypothyroidism, was associated with increased risk of SGA [7,8]. Similarly, in a prospective study of U.S. women, Männistö et al. reported a positive association of hypothyroidism and risk of preterm birth [27], which is consistent with findings of several other studies [28,29]. In our study of Finnish women, we observed an inverse association of Tg and SGA, but no association with preterm birth. TSH was not associated with either outcome, which was somewhat expected given that nearly all women were within the normal range.

While our findings for iodine are somewhat difficult to compare directly with prior studies due to our use of serum versus urine concentrations, the observed elevated risk of preterm birth associated with higher levels of serum iodide in our data are compatible with the possibility of a true U-shaped relationship observed in several previous studies, with both high and low levels of iodine being associated with increased risk of preterm birth. However, because the women who comprised our study population were only mildly iodine insufficient [3], and the prevalence of severe deficiency is low, our results may only capture the right side of this U-shaped relationship. Some data suggest that Tg levels may be sensitive indicators of iodine status among children and pregnant women [24,30], with a U-shaped relationship between Tg and UIC. Thus, the inverse association for SGA observed in analyses comparing high versus low Tg may be explained, at least in part, by mild iodine insufficiency among women with Tg in the lowest quartile. Additional explanations for conflicting findings of previous research and ours may relate to other underlying nutritional deficiencies (e.g., zinc or iron) present in other countries, bioavailable iodine in soil, and genetic factors, as well as differences in the underlying risk of obstetrical complications across study populations [3,31,32].

Potential biological mechanisms to explain our observed positive associations of serum iodide with preterm birth and inverse associations of Tg with SGA are somewhat unclear. During periods of iodine deficiency, TSH, which is secreted from the pituitary gland, increases and stimulates uptake of circulating iodine by thyrocytes in the thyroid [1]. The iodination of Tg produces T_3_ and T_4_, which are essential for fetal central nervous system and skeletal development. When iodine deficiency is severe (UIC <50 µg/L), the production of T_3_ and T_4_ is impaired, and circulating levels of the thyroid hormones decrease. Paradoxically, when iodine intake is excessive (defined as UIC >500 µg/L), a transient decrease in T_3_ and T_4_ also occurs, known as the Wolff–Chaikoff effect [33]. In normal, healthy individuals, this effect lasts only a few days; however, fetuses <36 weeks’ gestation cannot escape this effect, resulting in fetal hypothyroidism [34]. Although this phenomenon is not well understood, it is possible that high levels of maternal serum iodide may induce this decrease in fetal thyroid hormones, which in turn, could have implications for fetal skeletal development, placentation, and preterm delivery [33].

Strengths of our study include the population-based, nested case-control design, which ensures minimal impact of selection and recall biases, and improves generalizability to the source population. In addition, our study population included women who were mildly iodine-insufficient, but with relatively low prevalence of other nutritional deficiencies that might otherwise confound the associations between iodine status and preterm birth and SGA [3]. Furthermore, our study is the first to evaluate associations of preterm birth and SGA with serum iodine, a measure of iodine hypothesized to be more stable than urinary iodine concentrations over time. Single spot urinary iodine, which was used to assess iodine status in all previous studies on this subject, exhibits considerable intraindividual variation owing to urine dilution, dietary intake, circadian rhythm, season, and other factors [14,15,16]. Because of this, UIC is considered an acceptable biomarker of iodine status for whole populations, but not for individuals [35]. Serum iodine on the other hand, is less sensitive to recent dietary intake and may better reflect individual iodine status over longer periods of time [19], possibly reducing misclassification of exposure.

Our study also has limitations. First, we were limited to a single serum iodide measurement, rather than longitudinal iodine measurements over the course of the pregnancy. Serum iodide decreases during pregnancy, and as such, some women may have developed iodine deficiency later in pregnancy. Additionally, serum iodide reference ranges have not been established in this population, making it difficult to compare with prior research and World Health Organization iodine recommendations established for pregnant women. We also cannot rule out potential confounding by unmeasured factors such as dietary patterns, chronic thyroid conditions or iodine supplementation initiated after blood collection. Residual confounding by measured factors such as smoking and socioeconomic status is also possible, but the similarity between unadjusted and adjusted estimates suggest that this is an unlikely source of substantial bias.

## 5. Conclusions

In conclusion, we found that in a mildly iodine insufficient population of Finnish women, higher-serum iodide was positively associated with risk of preterm birth, but was not associated with risk of having an SGA infant. High levels of Tg were inversely associated with risk of SGA. In light of our findings, it appears that mild iodine insufficiency is unlikely to be a substantial contributor to preterm birth and SGA in Finland. Findings of increased risk of preterm birth associated with high-serum iodide warrant further investigation.

## Figures and Tables

**Table 1:** Maternal characteristics at blood draw according to preterm birth and small for gestational age cases and controls in the Finnish Maternity Cohort and Medical Birth Register, 2012–2013.

	Preterm Birth			Small for Gestational Age		
Characteristic ^1^	Controls (*n* = 242)	Cases (*n* = 208)	*P*-value ^2^	Controls (*n* = 241)	Cases (*n* = 209)	*P*-value ^2^
Maternal age (years)	29.5 (5.3)	29.8 (5.5)	0.49	29.5 (5.3)	29.7 (5.6)	0.70
Body mass index (kg/m^2^)	24.6 (4.4)	25.3 (6.0)	0.75	24.8 (4.9)	23.6 (5.1)	<0.001
Gravidity	1.4 (1.8)	1.4 (1.8)	0.80	1.4 (1.8)	1.3 (2.2)	0.03
Parity	1.1 (1.6)	0.9 (1.4)	0.19	1.1 (1.6)	0.8 (1.5)	0.01
Gestational age at screening (weeks)	10.9 (2.9)	10.8 (3.0)	0.89	10.9 (2.9)	11.4 (4.0)	0.12
Gestational age at birth (weeks)	39.7 (1.1)	34.1 (2.7)	<0.01	39.6 (1.5)	38.4 (2.9)	<0.001
Iodide (ng/mL)	28.1 (28.5)	32.8 (31.1)	0.02	28.2 (28.3)	27.7 (28.2)	0.82
Thyroglobulin (ng/mL)	29.6 (29.5)	31.5 (63.3)	0.36	29.9 (29.6)	27.9 (32.9)	0.05
Thyroid stimulating hormone (mIU/L)	1.2 (0.8)	1.3 (2.4)	0.25	1.2 (0.85)	1.4 (2.15)	0.68
Nulliparous	105 (43.4)	108 (51.9)	0.07	103 (42.7)	120 (57.4)	<0.01
Smoking status			0.33			0.03
Nonsmoker	200 (82.6)	164 (78.8)		199 (82.6)	155 (74.2)	
Smoker	37 (15.3)	35 (16.8)		37 (15.4)	52 (24.9)	
Unknown	5 (2.1)	9 (4.3)		5 (2.1)	2 (1.0)	
Socioeconomic status			0.32			0.11
Blue-collar	30 (12.4)	31 (14.9)		31 (12.9)	30 (14.4)	
Lower white-collar	64 (26.4)	60 (28.8)		62 (25.7)	50 (23.9)	
Upper white-collar	28 (11.6)	29 (13.9)		28 (11.6)	34 (16.3)	
Entrepreneur	10 (4.1)	4 (1.9)		10 (4.2)	3 (1.4)	
Student	25 (10.3)	12 (5.8)		24 (10.0)	10 (4.8)	
Other/unknown	85 (35.1)	72 (34.6)		86 (35.7)	82 (39.2)	
Diagnosed thyroid disease	0 (0)	7 (3.4)	<0.01	0 (0)	1 (0.5)	0.28
Chronic hypertension	3 (1.2)	10 (4.8)	0.024	3 (1.2)	4 (1.9)	0.71
Preeclampsia	3 (1.2)	30 (14.4)	<0.01	4 (1.7)	16 (7.7)	<0.01
Gestational hypertension	6 (2.5)	11 (5.3)	0.12	6 (2.5)	11 (5.3)	0.14
Type I or type II diabetes	1 (0.4)	13 (6.3)	<0.01	2 (0.8)	0 (0)	0.50
Gestational diabetes	22 (9.1)	38 (18.3)	0.02	23 (9.5)	23 (11.0)	0.64
Marital status			0.41			0.15
Married or cohabiting	215 (88.8)	176 (84.6)		211 (87.6)	177 (84.7)	
Single or widowed	26 (10.7)	31 (14.9)		30 (12.5)	29 (13.9)	
Unknown	1 (0.4)	1 (0.5)		0 (0)	3 (1.4)	

^1^ Values are means (SD) for continuous data and N (%) for categorical data. ^2^ *P*-values were estimated using t-tests for continuous data and χ^2^ tests for categorical data. Equivalent non-parametric tests were used where appropriate.

**Table 2:** Unadjusted and adjusted odds ratios (ORs) and 95% confidence intervals (CIs) for preterm birth according to maternal serum iodide, thyroglobulin, and thyroid stimulating hormone in the Finnish Maternity Cohort and Maternal Birth Register, 2012–2013 ^1,2^.

Biomarker	Cases: Controls	Median	Unadjusted OR (95% CI)	Adjusted ^3^ OR (95% CI)
Iodide (ng/mL)				
Quartile (Q)1	38:60	3.4	0.75 (0.46–1.22)	0.76 (0.46–1.26)
Q2 + Q3	102:121	20.3	1 (referent)	1 (referent)
Q4	68:61	59.3	1.32 (0.86–2.04)	1.22 (0.78–1.93)
Log(iodide)	208:242		1.22 (1.04–1.42)	1.19 (1.02–1.40)
Log(iodide) ^4^	132:210		1.29 (1.07–1.57)	1.29 (1.06–1.58)
Thyroglobulin (ng/mL)				
Q1	59:59	7.7	1 (referent)	1 (referent)
Q2	55:60	17.4	0.91 (0.55–1.53)	0.83 (0.49–1.41)
Q3	37:60	26.7	0.62 (0.36–1.07)	0.59 (0.34–1.04)
Q4	57:60	52.1	0.95 (0.57–1.58)	0.88 (0.51–1.50)
Log(thyroglobulin)	208:239		0.91 (0.76–1.08)	0.87 (0.73–1.05)
TSH (mIU/L)				
Low	5:11	0.04	0.51 (0.18–1.50)	0.57 (0.19–1.70)
Normal	196:217	1.04	1 (referent)	1 (referent)
High	7:11	3.5	1.14 (0.32–3.95)	1.17 (0.31–4.38)
Log(TSH)	208:239		0.99 (0.82–1.20)	0.97 (0.80–1.19)

^1^ ORs and 95% CIs were estimated using logistic regression. ^2^ Data are missing for 3 controls. ^3^ Multivariable models are adjusted for maternal age, maternal body mass index, socioeconomic status, smoking status, parity, and marital status. ^4^ Analyses excluding women with conditions indicated for preterm birth (i.e., preeclampsia, chronic hypertension, gestational hypertension, gestational diabetes, pre-existing diabetes, thyroid disease)

**Table 3:** Unadjusted and adjusted odds ratios (ORs) and 95% confidence intervals (CIs) for small for gestational age according to maternal serum iodide, thyroglobulin, and thyroid stimulating hormone in the Finnish Maternity Cohort and Maternal Birth Register, 2012–2013 ^1,2^.

Biomarker	Cases: Controls	Median	Unadjusted OR (95% CI)	Adjusted ^3^ OR (95% CI)
Iodide (ng/mL)				
Quartile (Q)1	52:60	3.3	1.05 (0.85–1.14)	1.01 (0.68–1.79)
Q2 + Q3	99:120	19.3	1 (referent)	1 (referent)
Q4	58:61	59.3	1.15 (0.74–1.80)	1.28 (0.79–2.08)
Log(iodide)	209:241		0.99 (0.85–1.14)	1.01 (0.86–1.18)
Log(iodide) ^4^	162:206		0.91 (0.77–1.07)	0.91 (0.76–1.09)
Thyroglobulin (ng/mL)				
Q1	75:58	8.3	1 (referent)	1 (referent)
Q2	50:61	17.7	0.63 (0.38–1.05)	0.52 (0.30–0.89)
Q3	36:59	28.2	0.47 (0.27–0.81)	0.41 (0.23–0.72)
Q4	45:60	52.3	0.58 (0.35–0.97)	0.45 (0.25–0.79)
Log(thyroglobulin)	206:238		0.84 (0.71–0.99)	0.78 (0.65–0.94)
TSH (mIU/L)				
Low	10:11	0.04	1.04 (0.43–2.50)	1.11 (0.44–2.79)
Normal	193:221	1.4	1 (referent)	1 (referent)
High	5:6	4.1	0.95 (0.29–3.18)	0.56 (0.14–2.22)
Log(TSH)	208:238		1.05 (0.88–1.26)	1.04 (0.86–1.26)

^1^ ORs and 95% CIs were estimated using logistic regression. ^2^ Data are missing for 3 controls. ^3^ Multivariable models are adjusted for maternal age, maternal body mass index, socioeconomic status, smoking status, parity, and marital status.^4^ Analyses excluding women with conditions indicated for preterm birth (i.e., preeclampsia, chronic hypertension, gestational hypertension, gestational diabetes, pre-existing diabetes, thyroid disease)

## Appendix A

The methods used to measure serum iodide have been described previously by Bell et al. [20]. After samples were thawed at room temperature and vortex mixed for 30 s, 200 μL were transferred to polypropylenes tubes. For sample pretreatment, an internal standard mixture of 40 ng/mL of Cl^18^O_4_- and 4 μg/mL of S^13^CN- was added and mixed. After the standard was thoroughly mixed, 20 μL of acetic acid (HAc, 5% in water) and 10 μL of ascorbic acid solution (AA, 2.5 mg/mL in water) were added, mixed, and incubated for 15 min in an incubator shaker at 37 °C (100 rpm). Following this, 100 μL of tetramethylammonium hydroxide (TMAH) (2.5 weight % solution in water), mixed the solution, and then digested in an oven at 90 °C for 2.5 h. Samples were then cooled to room temperature, after which we added 115 μL of water and 30 μL HAc, and then were mixed and centrifuged for 10 min at 5000 rpm. Finally, 500 μL of the final sample supernatant was transferred to amber vial for high-performance liquid chromatography-triple quadrupole mass spectrometry (HPLC–MS/MS).

An Alliance 2695 high-performance liquid chromatograph (HPLC) coupled with a Micromass Quattro LC tandem mass spectrometer (MS/MS; Waters Corporation, Milford, MA, USA) was used for sample analysis. Micromass MassLynx 3.5 software was used for data acquisition and quantification. The IonPac AS-21 column (guard column; 50 mm × 2 mm, analytical column; 250 mm × 2 mm, Dionex, Sunnyvale, CA, USA) was used to separate iodide. Identification and quantification of ^18^O-labeled-perchlorate, ^13^C-labeled-thiocyanate, and iodide was performed using electrospray negative ionization (ESI-) and multiple-reaction monitoring (MRM) mode at 107 (^35^Cl^18^O4-) > 89 (^35^Cl^18^O3-); 59 (S^13^CN-) > 59 (S^13^CN-), and 127 (^127^I-) > 127 (^127^I-).

A number of quality control checks were performed. A matrix matched calibration standard with a range of concentrations from 0.02 to 100 ng/mL of iodide was used for each 100-sample batch. Calibration curves had regression coefficients of >0.99. Each calibration standard had internal standards (Cl^18^O4- and S^13^CN-) spiked into them at 2 ng/mL and 20 ng/mL. Both internal standards had average recoveries of 70%. Estimated limits of quantification for iodide in blood sera was 0.25 ng/mL. Each 100-sample batch included a procedural blank, a matrix blank, a duplicate, standard reference material, and matrix spike of 25 ng/mL iodide. In analyses, no iodine was detected in procedural blanks, <2 ng/mL of iodide was detected in matrix blanks, 90%–111% of iodine was recovered in standard reference material, and 101%–122% of iodine was recovered in spiked serum matrices. No carry-over was detected from water blanks injected every 20–25 samples. To measure instrumental drift, a mid-point calibration standard was injected in every 10 h as initial calibration verification (ICV) to monitor for drift in instrumental response.
